# The needed link between open science and science diplomacy—A Latin American perspective

**DOI:** 10.3389/frma.2024.1355393

**Published:** 2024-06-06

**Authors:** Reina Camacho Toro, Luz M. Cumba Garcia, Laura A. Galvis, Luisa F. Echeverría-King, Branislav Pantović, Claudia Alarcón-López, Verónica Rossana Suarez, Pedro Figueroa, Ivonne Torres-Atencio, Claudia Widmaier, Tatiana Rodrigues Fraga, Susan Benavides

**Affiliations:** ^1^Laboratoire Physique Nucléaire et Hautes Energies (LPNHE), IN2P3/CNRS, Paris, France; ^2^Science Diplomacy Network in Latin America and the Caribbean (DiploCientifica), Santiago, Chile; ^3^American Association for the Advancement of Science (AAAS), Science & Technology Policy Fellowship, Washington, DC, United States; ^4^Inter-American Institute for Global Change Research (IAI), Science, Technology, and Policy (STeP) Fellowship, Montevideo, Uruguay; ^5^Vice Presidency for Research, Extension and Innovation, Universidad Simón Bolívar, Barranquilla, Colombia; ^6^International Relations Office, Universidad Nacional de Río Negro, Viedma, Argentina; ^7^School of Engineering, École Polytechnique Fédérale de Lausanne (EPFL), Lausanne, Switzerland; ^8^Learning Science & Higher Education, Eidgenössische Technische Hochschule Zürich (ETHZ), Zurich, Switzerland; ^9^Agencia Uruguaya de Cooperación Internacional (AUCI), Montevideo, Uruguay; ^10^Pharmacology Department, Faculty of Medicine, University of Panama, Panama City, Panama; ^11^Instituto de Investigaciones Científicas y Servicios de Alta Tecnología de Panamá AIP, Panama City, Panama; ^12^Organization for Women in Science for the Developing World, Trieste, Italy; ^13^Department of Immunology, Institute of Biomedical Sciences, University of São Paulo, São Paulo, Brazil; ^14^Office of the Vice-President for Research and Outreach, Universidad de América, Bogotá, Colombia

**Keywords:** science diplomacy, open science, Latin America (LATAM), Global South, international collaboration, scientific cooperation

## Abstract

The relevance of science diplomacy and open science in today's world is undeniable. Science diplomacy enables countries to jointly address pressing global challenges, such as climate change, pandemics, and food security. Open science, promoting accessible and transparent research, plays a pivotal role in this context. Nevertheless, the degree of openness is subject to specific circumstances, contingent upon varying factors, including local knowledge and resources. Latin America has not only been at the forefront of pioneering open access strategies, making it an interesting case to study, but it has also shown a tangible interest in using science diplomacy. Our research employs a mixed-methods approach, incorporating a quantitative survey involving 50 organizations and initiatives dedicated to promoting open science in Latin America, along with two qualitative focus group studies. Our primary objective is to assess if and how these entities use science diplomacy to achieve their objectives. Non-policy entities were prioritized due to their institutional stability in the region. We highlight successful strategies and delve into the existing barriers hindering the full implementation of open science principles. Our research aims to enhance collaboration between these organizations and policy and decision-makers by providing a set of recommendations in that direction. By shedding light on the current landscape and dynamics of open science in Latin America, we aspire to focus on science diplomacy, facilitate informed decision-making, and formulate policies that further propel the region along the path of openness, collaboration, and innovation in scientific research.

## 1 Introduction

The convergence of science diplomacy and open science holds the potential to unite nations in addressing global challenges by enhancing the credibility and impact of research findings. The COVID-19 pandemic highlighted the need for universal access to scientific progress and cooperation in science, technology, and innovation (STI). Open science ensures access to scientific knowledge and educational resources, promoting a culture where knowledge access is a right for all. Science diplomacy connects international actors, such as scientists, policymakers, and international organizations, to address global challenges and strengthen scientific capacities (Gittens et al., 2021). While the precise definition of science diplomacy has evolved over the last 15 years, these changes have mirrored shifts in global politics. This underscores the vital role of international actors in countering national sentiments and fostering rational and diligent approaches to global challenges (Rungius and Flink, 2020).

We advocate for responsible and accessible knowledge production in Latin America (LATAM), tailored to regional needs while contributing to international and global discussions. This foundation is crucial for tackling complex challenges that demand cross-sectoral and international collaboration. For LATAM, science diplomacy and open science are ongoing processes that require reflection on our current practices.

This qualitative-quantitative study aims to explore the utilization of science diplomacy strategies by organizations and initiatives promoting open science in the region. We specifically targeted the ones that wield influence in the development of science policy, albeit indirectly. Given the high rate of personnel change in governmental institutions, non-policy actors were prioritized due to their institutional stability in the region. Non-policy actors refer to organizations, universities, research institutions, libraries, NGOs, publishing services, and other actors which advocate for policy making/change. Our analysis seeks to provide recommendations for these organizations and policy and decision-makers to enhance their synergy, thereby fostering the democratization of knowledge and sustainable development at both global and local levels.

## 2 Science diplomacy from Latin America's perspective

The term “science diplomacy,” though relatively recent, has rapidly evolved since its formal definition in 2010 as the convergence of science, technology, innovation, and international affairs (The Royal Society and AAAS, 2010). Over the past decade, science diplomacy has transformed into a multidimensional practice, fostering collaboration among diverse stakeholders including governments, international organizations, scientific communities, non-governmental organizations (NGOs), the private sector, and civil society. These collaborations, whether serving national or international interests, have become crucial for addressing complex global challenges (Pantovic and Michelini, 2018). Science diplomacy is a concept that varies depending on political contexts, the state of scientific systems, and specific objectives. It encompasses diverse topics, goals, and approaches, making it challenging to define universally. Broadly, science diplomacy is seen as the intersection between actions in science and foreign policy, serving as a bridge between the two realms (Rungius and Flink, 2020). Alternatively, it can be viewed as the interaction between diplomacy and science to address national needs, cross-border interests, or global challenges (Gluckman et al., 2017). While one definition emphasizes the constructivist aspect of the concept, the other focuses more on providing scientific advice in foreign affairs.

The LATAM region has demonstrated a growing interest in science diplomacy, evident in both academic and foreign policy agendas. However, its precise conceptualization in the region remains a work in progress (Vera and Echeverría-King, 2022; Echeverria-King et al., 2023). LATAM countries exhibit significant diversity in their scientific and political landscapes, leading to differing approaches to science diplomacy. These variations arise from differences in political cultures, foreign policy objectives, and decision-making processes, influenced by policymakers' skills and academic backgrounds (Ayala et al., 2023).

Science diplomacy in the LATAM region faces the challenge of aligning with global and national needs, requiring diplomacy adjustments and skills development. It is important to recognize that science diplomacy's prominent activities and discourses are often driven by the Global North, which may not suit the region's unique problems. Actions promoted by the Global North may have unintended adverse effects in the LATAM region, as they frequently overlook and marginalize diverse perspectives that fall outside their established frameworks (Seitz, 2010). Therefore, decision-makers should consider local knowledge systems and practices to address regional issues effectively. Although this is especially critical when addressing complex, transdisciplinary challenges, there is a gap between the demand for training and the practical implementation of science diplomacy in the LATAM region (Echeverria-King et al., 2023).

While the LATAM region lacks a common approach to science diplomacy, its diverse nature can be observed through three main facets: (1) bilateral and multilateral collaboration, involving diplomatic interactions and collaborations between two or more countries on STI issues (Frech et al., 2018; López-Vergès et al., 2021); (2) policy and decision-making, focusing on how scientific evidence, expertise, and advice contribute to shaping policies, regulations, and agreements (Garton et al., 2021; Soler, 2021); and (3) public engagement and international outreach, emphasizing the importance of science communication, dissemination of scientific results, public outreach, and government engagement with both policy and non-policy actors (Hajdu and Simoneau, 2020; Pulido-Salgado and Castaneda Mena, 2021; Massarani and de Oliveira, 2022; Echeverría-King et al., 2024). These facets can be linked to the “access,” “promotion,” and “influence” framework proposed by Flink and Schreiterer (2010). This study uses this framework, driven by three main questions. First, it examines whether non-policy actors participating in the study are securing access to resources through bilateral or multilateral collaborations to enhance the open science ecosystem at the national, regional, or global levels. Second, it explores whether these actors promote national or regional open science work in global discussions. Third, it investigates their interactions with society, policymakers, and decision-makers at national or regional levels, as well as their participation in global debates regarding the adoption of open science and its principles. This framework offers insights into overarching strategies, enabling us to engage with actors operating beyond national borders. It is essential to recognize that these dimensions are interconnected and often reinforce each other, contributing to the region's soft power (Nye, 2004). However, caution is advised against overly politicized agendas, as science diplomacy aimed solely at serving national objectives may clash with international interests (Ruffini, 2020).

## 3 Open science from Latin America's perspective

Science is pivotal in achieving the “Sustainable Development Goals” and the “2030 Agenda” as it offers solutions to contemporary challenges, fosters interdisciplinary collaboration, and focuses on problem-solving initiatives. To benefit society at large, science must evolve to become more collaborative, transparent, accessible, equitable, responsible, and inclusive. These are fundamental tenets of open science, a concept that has garnered increasing attention in recent years.

The United Nations Educational, Scientific and Cultural Organization (UNESCO)'s Recommendation on Open Science (UNESCO, 2021) defines open science as an inclusive concept that aims to make scientific knowledge available to all, fostering scientific collaboration and information exchange for the betterment of science and society. Open science can be explained as an umbrella term aiming to remove barriers to accessing scientific processes and outputs, including but not limited to access to publications, research data, open-source software, open collaboration, open hardware, open peer review, educational resources, citizen science and even research crowdfunding (FOSTER, n.d.).

Open science is gaining importance in the LATAM region, with varying policies influenced by national priorities and funding availability. Efforts are underway to promote open science, with instances of political dialogue facilitated through regional blocs like the European Union – Latin America and Caribbean (EU-LAC) and the Southern Common Market (MERCOSUR). The EU-CELAC Joint Initiative for Research and Collaboration (JIRI) exemplifies cooperation in science and research, emphasizing access to research infrastructures and data sharing (RESINFRA Project, 2018). In MERCOSUR, discussions focus on regional, national, and institutional policy changes, as well as designing training programs for researchers and evaluation strategies (MEC Uruguay, 2022).

Digital repositories have been a priority for data access and open-access publishing policies. Several LATAM countries have approved national legislation to house government-funded research results in open-access digital repositories (International Science Council, 2018). In 2012, various science and technology agencies from LATAM countries initiated The Federated Network of Institutional Repositories of Scientific Publications (LA Referencia) to bolster open access policies, ensure unrestricted access to publicly funded research, and enhance the visibility of scientific contributions in the LATAM region. LA Referencia, the paramount regional initiative for governing and coordinating open-access policies, is managed by the Latin American Cooperation of Advanced Networks (RedCLARA) and includes 12 member countries. LA Referencia collaborates closely with two key organizations, the Open Access Infrastructure for Research in Europe (OpenAIRE) and the Confederation of Open Access Repositories (COAR). Together, they promote knowledge exchange through open science repositories, share best practices, and facilitate international collaboration through research and policymaking (LA Referencia, 2012). Other open–access repository platforms in the region include the Regional Online Information System for Scientific Journals in Latin America, the Caribbean, Spain, and Portugal (Latindex), the Scientific Electronic Library Online (SciELO), and the Diamond Open Access Scientific Journal Network (Redalyc), contributing to open science dissemination (Open Access Resources for International Area Studies, 2020). In open access initiatives, LATAM also collaborates with other regions, such as South Africa through platforms like SciELO-South Africa (Schöpfel, 2015).

However, open science in the LATAM region must also navigate the challenge of aligning global expectations with local realities. The region's diverse capacities in science, technology, and innovation, coupled with variations in funding, infrastructure, and resource accessibility, highlight the importance of tailored approaches to suit particular circumstances.

## 4 Connections between science diplomacy and open science

The global adoption of open science practices and collaborative tools based on digital technologies like Zenodo, arXiv, and OpenReview, among many others, will involve the development of policies and coordination with governments and international organizations. These efforts aim to establish standards and legal frameworks for exchanging scientific information openly, and to create formal spaces for international stakeholders to collaborate on research, data management, and sharing. This transition to open science can be greatly facilitated by leveraging diplomatic capabilities to bridge national and international interests. Additionally, creating platforms for multi-level discussions and providing advice to policymakers can help navigate the complexities of this transition.

The interaction between open science and science diplomacy is facilitated by international instruments that work to make science more accessible and equitable. Examples include the Budapest Open Access Initiative Declaration (2002), Berlin Declaration on Open Access to Knowledge in the Sciences and Humanities (2003), and Bethesda Statement on Open Access Publishing (2003). Each of them advocates for open access to scientific information and knowledge (Declaración de Acceso Abierto, 2020).

As this shift unfolds, it will significantly impact the work of science diplomats and policymakers. New open-access partnerships will emerge, and scientific knowledge will become more accessible, empowering decision-making processes. Despite facing limitations in the international arena due to resource constraints and their position in the Global South, Latin American states have the potential to overcome these challenges (Ayala et al., 2023). By harnessing the synergy between science diplomacy and open science, they can address governance obstacles. This represents a dual challenge, requiring proactive measures to both navigate governance hurdles and tackle pressing issues head-on.

The Using Science for/in Diplomacy for Addressing Global Challenges (S4D4C) Project, funded by the European Union (Mayer, 2020) explored, back in 2020, the relationship between science diplomacy and open science in the light of European efforts in both areas. The study emphasizes the need for diplomatic skills in the open science ecosystem and vice versa, highlighting the potential benefits of international scientific cooperation and innovation.

Within the LATAM region, science diplomacy has the potential to play an important role in further enhancing open-access repositories and shaping new policies and regulations to promote open science. Both international and regional collaborations facilitate scientific discovery, technology transfer, and capacity building. Examples of such collaborative networks include the Latin American Council of Social Sciences (CLACSO) (CLACSO, 2024) at the regional level and the Global Research Council (Global Research Council, 2023) at the international level. A developed relationship between science diplomacy and open science can contribute to the advancement of international scientific cooperation, innovation, and the democratization of knowledge on a global scale (EU-LAC Interest Group, 2023).

LATAM stands out as a trailblazer in open–access publications, serving as a model for other regions like SciELO-South Africa. While the open access model developed in the region is well-established, it often lacks recognition, especially in scientific assessment systems. Consequently, researchers in LATAM frequently resort to paying article processing charges (APCs) to publish with commercial publishers abroad. Science diplomacy presents an opportunity to forge global alliances promoting open–access models and moving away from APCs. Moreover, it can bolster Latin America's participation in coalitions focused on various open science aspects, including data access and research assessment reform.

Countries like Argentina, Brazil, Chile, Colombia, Costa Rica, Mexico, and Peru have made significant strides in developing open–access and research data policies and initiatives. However, the heterogeneous nature of funding, governmental stability, infrastructure, and resource accessibility has hindered consistent implementation at the institutional level. Throughout this process, non-policy actors such as universities, research institutions, libraries, NGOs, and publishing services have played a pivotal role of influence at the local, regional, and global levels and also at grounding the initiatives. We aimed to explore whether these non-policy actors engage in science diplomacy activities to achieve their goals, to highlight successful strategies as well as the main barriers they encounter when promoting open science.

## 5 Methodology

### 5.1 General purpose of the study

The study sought to identify and characterize organizations and initiatives promoting open science in the LATAM region and assess their use of science diplomacy to achieve their goals. It also aims to provide recommendations for these organizations, public policy, and decision-makers to enhance the synergy between science diplomacy and open science processes in the region. The research focused solely on non-policy actors to explore how organizations without direct involvement in policy-making utilize tools of science diplomacy. Given the elevated rate of personnel changes in governmental bodies, non-policy actors were prioritized due to their higher institutional stability in the region.

### 5.2 Design and procedure

The study employed a mixed research design, which provides a holistic perspective that enriches the interpretation of the results and facilitates a complementary approach according to the research objectives. This combination of methodological approaches offers greater validity and reliability in addressing the complexity inherent in the initial explorations, allowing researchers to obtain an accurate and contextualized view of the issues under study (Shorten and Smith, 2017). Data collection was carried out between July and October 2023.

For the quantitative analysis, a survey was distributed by email to 110 organizations and initiatives promoting open science within their activities in LATAM. Some organizations shared the survey with their mailing lists, expanding its initial intended outreach. The survey aimed to understand the status of open science in the region and identify potential science diplomacy actions undertaken by these organizations. The survey was divided into five sections: (1) sociodemographic analysis; (2) organization characteristics; (3) open science conceptualization; (4) science diplomacy initiatives; and (5) interaction with other stakeholders. The framework developed by Flink and Schreiterer (2010) was used to design the questions aiming to identify and characterize implicit science diplomacy strategies. The list of questions can be found in Appendix 1. In total, 286 responses were obtained. The exclusion criteria included individuals not in a position to represent an organization, policy actors, and those lacking a clear link to Latin America or open science. After filtering, 50 responses were retained for analysis. The organizations retained included NGOs/CSOs (18%), learned societies (6%), publishing and research services (10%), research infrastructure organizations (14%), research performing institutions (24%), higher education institutions (22%), and charitable organizations/trusts (4%).

For the qualitative analysis, two focus groups were conducted via the Zoom platform in October 2023, with the participation of three experts in open science in each group. Delegates from non-policy entities who completed the survey were prioritized. Participants (Table 1) provided informed consent for the research. After each focus group session, transcriptions were prepared, and the inputs were analyzed using the qualitative content analysis method. Categories and subcategories were created inductively, aligning with the research objectives and the gathered information. The research report incorporated quotes from the focus group discussions.

**Table 1:** Participants in the focus groups.

**Participant 1**	**Representative of the Open Science Institution of Ecuador**
Participant 2	Representative of the Open Science Institution of Ecuador
Participant 3	Representative of the Open Science Institution of Chile
Participant 4	Representative of the Open Science Institution of Argentina
Participant 5	Representative of the Open Science Institution of Brazil
Participant 6	Representative of the Open Science Institution of Mexico

## 6 Results

### 6.1 Survey results

**Figure 1:**
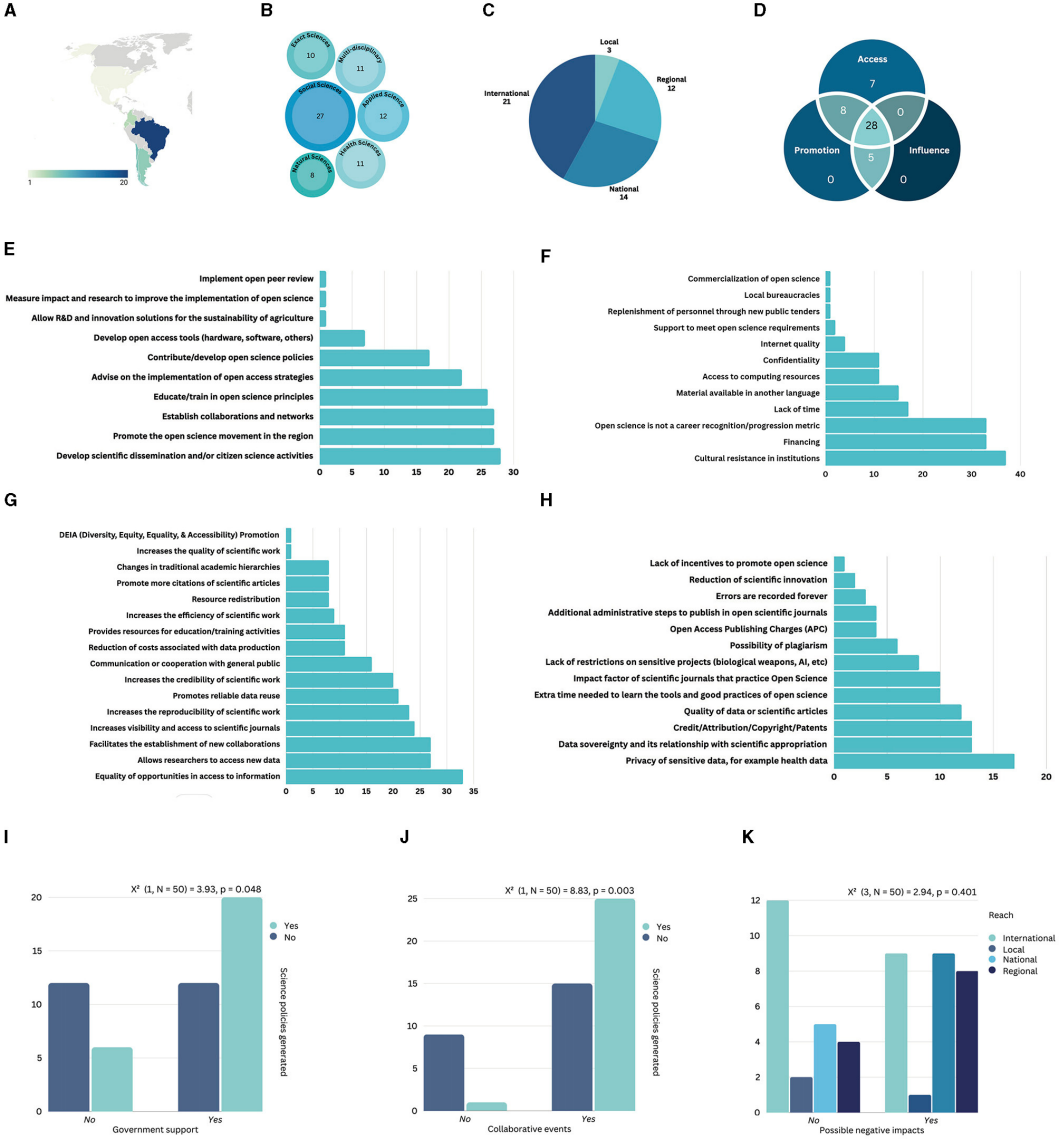
Summary of survey results. **(A)** Distribution of survey participants based on their countries of residence. Map generated with Datawrapper (www.datawrapper.de). **(B)** Scientific fields of participating organizations. **(C)** Geographic reach of participating organizations. **(D)** Types of engagement reported by participants, categorized as access, promotion, and influence according to Flink and Schreiterer (2010). **(E)** Stated objectives of the organizations as self-reported by the participants. **(F)** Barriers identified by participants in the implementation and development of open science. **(G)** Positive impacts of open science on research as reported by participants. **(H)** Negative impacts of open science on research as reported by participants. **(I)** Correlational analysis between open science policy generation and the organization's access to government support. **(J)** Correlational analysis between open science policy generation and engagement in collaborative events with other organizations. **(K)** Correlational analysis between the geographic reach of the organization and the perception of possible negative impacts reported by survey participants.

The survey participants exhibited a geographical bias in terms of their country of residence, with a predominant representation from Brazil, Chile, and Argentina, collectively constituting two-thirds of the responses (Figure 1A). The Andean Region was represented only by Colombia and Ecuador, and Costa Rica was the sole Central American contributor. Mexico and the United States of America had a single contribution each. The origin disparity in participants' countries raises questions about whether this reflects a survey sampling bias due to the sharing of the survey, or indicates regional variations in the prevalence of open science initiatives.

The surveyed organizations demonstrated a diverse range of fields, with nearly half of the responders indicating involvement in more than one area of interest. This multidisciplinary engagement underscores the widespread interest in open science across various fields. Importantly, the majority of organizations engaged in open science are not specialized in a particular topic. Considering the different areas, social sciences emerged as the most frequently mentioned (34%) (Figure 1B).

The scope of these organizations or initiatives is predominantly international (42%) or regional (24%) (Figure 1C), with over 25% having a local or national reach. The primary reported objectives of these entities are promoting and training in open science (Figure 1E). Moreover, 60% of organizations stated that advising on the implementation of open science strategies is one of their objectives, and 50% aimed to contribute to or develop open science policies (Figure 1E).

To understand the interactions of these organizations with other stakeholders, we inquired about their government support, collaboration with international organizations, and involvement in open science policies at the local, regional, or international levels. Interestingly, institutions organizing collaborative events with foreign organizations were significantly more likely to contribute to open science policies, as highlighted by a chi-squared test [$\chi_{\left(1,\text{N}=50\right)}^{2}$ = 8.83, *p* = 0.003] (Figure 1J). This underscores the pivotal role of international collaborative activities in facilitating an exchange of ideas and strategies to foster the open science agenda. Data also indicate a correlation between organizations that received government support and the organization's impact on open science policy, as indicated by a chi-squared test [$\chi_{\left(1,\text{N}=50\right)}^{2}$ = 3.93, p = 0.048] (Figure 1I). However, this effect is of borderline significance, implying that other factors also play crucial roles in policy influence. Additionally, organizations that have an international reach (i.e., working with organizations in countries outside LATAM) reported fewer negative perceptions of open science compared to their national and regional counterparts, as observed in Figure 1K. Although this difference was not statistically significant (*p* = 0.401), it suggests the potential mitigating impact of international engagement on negative perceptions of open science.

Next, we aimed to evaluate whether these organizations were implicitly employing science diplomacy strategies in pursuit of their open science objectives. To assess the engagement of these organizations in science diplomacy, we applied the Flink and Schreiterer (2010) framework categorizing the activities into “access,” “promotion,” or “influence” (Figure 1D). Among the 50 organizations, only two did not report any of these aims in their activities. Access emerged as the most common science diplomacy objective, with no organization solely engaged in promotion or influence. However, 56% were involved in all three categories. The prevalence of access aligns with the organizations' reported objectives, primarily focused on the dissemination, promotion, and training in open science. Reported initiatives aimed at ensuring access to resources for enhancing the country's and/or region's innovation and competitiveness capabilities included training, establishing international collaborations, access to hardware or software, research in open science, and repository development.

Finally, we surveyed the perceptions of these organizations around open science. The primary reported barrier to open science was cultural resistance within institutions (e.g., reluctance to incorporate new practices or processes by any actor of the system), closely followed by financial constraints (e.g., fees to publish in APC journals), and a lack of recognition as a career metric (e.g., contributions to open science or using open science principles are not taken into account to grant funds or promotions) (Figure 1F). The most commonly cited positive impacts of open science were enhanced access to new data and equal opportunities for information access. Other positive impacts included increased visibility of scientific journals, heightened reproducibility of scientific work, and the establishment of new collaborations (Figure 1G). It is also important to report that 54% of respondents from the surveyed organizations expressed concerns about the potential negative impacts of open science on their scientific work. These concerns encompassed issues such as the privacy of sensitive data, data sovereignty, appropriation of scientific work, and the quality of scientific data, among others (Figure 1H).

### 6.2 Focal groups input

In general, all participants agreed that the open science movement in the LATAM region is in a developing phase, emphasizing the need for increased awareness, international and regional cooperation, and educational efforts. They also underscored the region's long standing tradition of open access to publications and the establishment of open-access repositories. Additionally, there was consensus on the necessity of addressing other facets of the open science framework, such as open data management (participants 1, 2, 3, and 5), equitable funding (participants 4 and 6), citizen science involvement (participant 3), and capacity building and training (all participants).

While initially unfamiliar with the concept, three out of the six participants quickly grasped the potential of science diplomacy to enhance international cooperation in science and technology to address local and regional needs. They emphasized the importance of balancing local and international perspectives in the transition toward open science in LATAM. Participant 4 pointed out that “we have a perspective of local and regional development, but always connected to the global, the international, not completely detached (...) building this kind of trust makes open science more visible, a bit more international.” They cited specific regional needs such as training materials in Spanish, stable funding mechanisms, and recognition systems to accelerate the transition toward open science. Participant 3 mentioned that “we work to achieve a dialogue between organizations in Europe and Latin America and the Caribbean, where we start from the fact that each can learn from the other (…) but also a dialogue between institutions in Latin America, sometimes we do not realize that within the region there are also institutions that are very advanced and that we can really learn from those institutions and that is very important because I mentioned the issue that the challenges are global, but often the solutions are regional because the reality is very different, so global challenges, local answers.” Participant 6 underscored the significance of diplomatic efforts to foster more inclusive scientific collaboration, advocating for the inclusion of local actors in global discussions.

Some successful examples of science diplomacy involving these organizations were mentioned during the interviews, such as the CERN-NASA Open Science Summit 2023. This event concluded with statements translated into five languages (Spanish, French, German, Chinese, and Arabic), advocating for a transition to a more open, participatory, equitable, robust, and sustainable research ecosystem (CERN/NASA, 2023). Additionally, UNESCO's global call for best practices in open science in 2021 resulted in the UNESCO Recommendation on Open Science. Participant 1 noted that global discussions have influenced the development of national policies, offering valuable lessons for countries without existing open science policies.

Regarding current barriers to open science in the region, Participant 1 noted that the current measurement of the quality of research has commercial implications, limiting collaboration and negotiation between researchers. Similarly, Participant 3 criticized economic barriers imposed by publishers, hindering the dissemination of quality information, especially in addressing global challenges. This gap between commercial interests and national needs is crucial and warrants special attention, particularly in the context of open science and science diplomacy. Participants 3, 4, and 6 pointed to the lack of stable funding as a major barrier, with Participant 1 also noting political instability as a factor affecting funding and policy development and implementation. It should be mentioned that some participants identified the challenge of involving diverse actors as a major barrier to open science. This is crucial to highlight, as science diplomacy relies on the engagement of a diverse range of actors to effectively carry out international activities, and this characteristic is considered a key aspect of science diplomacy. Participants have organized training and seminar cycles to showcase open science practices, with Participant 2 suggesting extending these efforts to involve other actors in the regional open science ecosystem, which could garner more support from policymakers.

When discussing additional good practices for promoting and advancing open science, Participant 3 noted: “Latin America and the Caribbean is the only region that currently does not have contemplated an open science cloud, which is a confederation of open science infrastructure.” This underscores the need to expand expertise in the region beyond open-access publications to include data and other aspects of open science. Research infrastructures typically involve various stakeholders and require multi-level negotiations covering standards, protocols, governance, cost sharing, and ownership.

Participant 4 emphasized the importance of “building communities (…) that are healthy to sustain open science.” This idea of community building is echoed by Participant 2, who emphasized the importance of alignment with other initiatives within their countries. Participant 2 considered that policies to protect scientific data, ensure repository infrastructure, and establish a proper methodology for data management would benefit open science researchers. In that regard, Participant 5 advocated for high-level support for open-access publications and underscored the importance of FAIR (findable, accessible, interoperable, and reusable) access (Wilkinson et al., 2016) to research data.

Participants also delved into the potential impact of open science on science diplomacy from their perspective. Participant 3 highlighted that we are in troubled times, and facing many global challenges. In this context, Participant 3 reflected on how the COVID-19 global emergency led to the sudden release of a high percentage of COVID-related publications. Participant 6 described open science as the democratization of the scientific process, both geographically and in terms of society, emphasizing the common goal of advancing knowledge and solving such global challenges through more inclusive and representative scientific collaboration.

Participant 3 highlighted the significant role of non-policy actors within the open science ecosystem, emphasizing their potential to inform and shape national policies from the ground up. They can provide valuable insights into practical aspects such as tool usage, data management planning, and necessary modifications to recognition and funding programs. Participant 6 expanded on this notion, noting that organizations operating across national borders hold influence over regional and global discussions. They have the ability to ground rules and policies in the specific realities of participants, considering factors like territorial differences, cultural nuances, and varying intellectual property regulations. Additionally, Participant 6 stressed the importance of involving local researchers in international projects to ensure relevance and effectiveness.

## 7 Recommendations

Based on insights from focus groups and research on the status of open science and science diplomacy in the LATAM region, the following recommendations are derived:

**For organizations promoting open science:**

- **Advocacy and lobbying:** Engage in discussions with governments and international organizations to emphasize the importance, challenges, and benefits of open science (see Section 6.1 paragraph 4; Section 6.2 paragraph 2, 5, 6, and 8).
- **Community building:** Establish and strengthen communities for sharing resources, best practices, and roadmaps tailored to local needs for the adoption of open science (see Section 6.2 paragraph 5 and 6).
- **Training**: In addition to training related to open science's technical aspects and good practices for researchers and academics, extending training to other actors within the open science ecosystem, such as science managers and policymakers, could amplify the impact of their activities. The adoption of open science practices takes time and requires institutional cultural changes and learning new tools (see Section 6.1 paragraph 6; Section 6.2 paragraph 4).

**For public policy and decision-makers:**

- **Scientific data, process, and outputs:** Develop policies to protect and define proper methodologies for scientific data and outputs management. Ensure that data is standardized, accessible, interoperable, and reusable (FAIR principles) (see Section 6.2 paragraph 1, 5, and 6).
- **Open science infrastructure:** Promote the creation and guarantee access to sustainable shared infrastructures needed for implementing open science practices, such as repositories and cloud services (see Section 6.2 paragraph 5).
- **Recognition and incentives:** Create policies for recognizing and incentivizing the use of open science practices in academia and research environments (see Section 6.1 paragraph 6).
- **Funding:** promote the allocation of more funding to support open science initiatives and practices (see Section 6.1 paragraph 6; Section 6.2 paragraph 1, 2, and 4).

## 8 Conclusions

The concepts of science diplomacy and open science share significant commonalities and synergies, as they share objectives of fostering international collaboration, addressing global challenges, and informing evidence-based policy formulation. Both practices involve collaboration between actors from the realms of science, policy, and politics, emphasizing the importance of international cooperation and a certain level of transparency to achieve their objectives. Moreover, both practices serve complementary functions by bridging the gap between different sectors. These shared foundational values highlight the imperative of global engagement in both science diplomacy and open science endeavors.

Despite their global origins, both science diplomacy and open science require tailored approaches when implemented in specific regions to maximize their effectiveness. This necessitates a comprehensive understanding of local conditions, proactive problem-solving to overcome obstacles, and adaptive strategies tailored to local contexts. Such an approach is crucial for ensuring the relevance and impact of both practices, as highlighted by insights from interviews.

This study involved the participation of 50 representatives from organizations promoting open science in LATAM. The study was limited to non-policy actors, including universities, NGOs, research institutions, and publishing services, as these institutions often fulfill needs left unanswered by the instability of governments and lack of continuity of state policies in the region. Participants note significant progress in regional open science, stressing the importance of policymakers working hand in hand with the scientific community to establish collaborative frameworks. Moving forward, fostering open science in the region will require finding a harmonious balance between local and global perspectives, highlighting the crucial role of diplomatic efforts to engage local stakeholders in broader global conversations. Furthermore, non-policy actors within the open science ecosystem, as identified in the study, can serve as essential grassroots contributors, grounding national policies and injecting local/regional viewpoints into global discourse. While all participants recognize the visibility of science through open science, the results underscore the need for an official commitment to transparently harnessing the power of science and access to knowledge to promote wellbeing, forming the nexus between science diplomacy and open science.

These commitments, made by regional organizations, typically involve establishing standards and frameworks, enhancing transparency, and fostering international engagement in STI endeavors tailored to address specific challenges and needs. This includes activities such as capacity building, policy formulation, and the negotiation of agreements by governments or intergovernmental bodies, aimed at advancing both the principles of open science and the objectives of science diplomacy simultaneously. Future research could further explore the impact of open science on science diplomacy and evidence-informed decision-making, contributing to a deeper understanding of their interplay, as well as expanding the scope to include policy actors and foreign representatives within the open science ecosystem.

The quantitative results highlighted “access” as the primary contribution of open science actors within the science diplomacy framework outlined by Flink and Schreiterer (2010). A significant portion (86%) of organizations reported seeking to guarantee access to resources to improve national or regional innovation and competitiveness through international collaborations. These collaborations primarily focused on promoting and training processes in open science, playing a crucial role in increasing access to knowledge and tools at national or regional levels, while also shaping perceptions of open science. Additionally, international promotion of national and regional open science best practices was highly cited (82%). This promotion effort was further evidenced in the focal groups, where participants shared their work and highlighted the region's longstanding tradition of open access to publications and the establishment of open-access repositories as exemplary in an international context. Interactions with society and policymakers, as an “influence” strategy regarding the adoption of open science and its principles, appeared as a secondary strategy (66%), evolving alongside expanding international collaborations and engagement with government institutions. Importantly, no organizations reported involvement in “influence” activities without engagement in “promotion,” and only five organizations reported engagement in “influence” and “promotion” without “access.” These findings highlight the interconnectedness of these dimensions and suggest potential pathways for open science organizations to become more active in “promotion” and “influence.”

Enhanced convergence of science diplomacy and open science practices can yield a tool that serves political objectives while contributing to academic and scientific development addressing global challenges. The meaning and purpose of this connection can vary for each political-scientific mission, covering diverse topics and approaches based on relevance, needs, and budget considerations. Despite the distinct perspectives of scientists, policymakers, and decision-makers, a flexible collaboration is essential for national and regional development, achieving common goals, and contributing to foreign policy efforts. This dynamic interaction facilitates a constructive review of international interaction models and global governance, fostering a mutually beneficial relationship between the realms of politics and science.

Our aspiration with this study extends beyond facilitating further research on the connection between science diplomacy and open science. We advocate for greater responsibility among policymakers, urging them to promote synergy between these practices to benefit the development of the LATAM region. In this context, we, the authors, find that the discussion points to the fact that science diplomacy and open science are viewed as mutually reinforcing tools. Their connection is recognized as an impetus for the development of science policies, improvement of national science ecosystems through (international) cooperation among different actors facing shared challenges, and spotlighting regional and national scientific achievements.

## Data availability statement

The anonymized raw survey data supporting the conclusions of this article will be made available by the authors upon reasonable request, while ensuring the protection of participants' identities.

## Ethics statement

Ethical approval was not required for the study involving humans in accordance with the local legislation and institutional requirements. The studies were conducted in accordance with the local legislation and institutional requirements. The participants provided their written informed consent to participate in this study.

## Author contributions

RCT: Conceptualization, Data curation, Formal analysis, Funding acquisition, Investigation, Methodology, Project administration, Supervision, Validation, Writing – original draft, Writing – review & editing. LCG: Conceptualization, Project administration, Supervision, Writing – original draft, Writing – review & editing. LG: Data curation, Formal analysis, Investigation, Methodology, Software, Validation, Visualization, Writing – original draft. LE-K: Conceptualization, Data curation, Formal analysis, Investigation, Methodology, Writing – original draft. BP: Data curation, Formal analysis, Investigation, Methodology, Validation, Writing – original draft, Writing – review & editing. CA: Formal analysis, Investigation, Visualization, Writing – original draft. VS: Writing – original draft, Data curation, Formal analysis, Methodology. PF: Conceptualization, Investigation, Methodology, Writing – original draft. IT-A: Conceptualization, Investigation, Methodology, Writing – original draft. CW: Investigation, Validation, Writing – original draft. TF: Investigation, Validation, Writing – original draft. SB: Writing – original draft.
