# Supplementary material for: The needed link between open science and science diplomacy—A Latin American perspective
Source: Front Res Metr Anal. 2024 Jun 6;9:1355393. doi: 10.3389/frma.2024.1355393 (PMC11187346; doi:10.3389/frma.2024.1355393)
Supplement: Supplementary file 1 [file Data_Sheet_1.docx]

**Appendix 1**

List of questions included in the quantitative survey:

**Section 1: Socio-demographic analysis**

1) Full name

2) Level of education

Undergraduate

Master

PhD

Other

3) Nationality

4) Country of residence

5) Name of the organization

6) Job within organization

Volunteer

Regular member

Member of the board

President

Consultant

Student

Teacher

Research staff

Administrative staff

Other

**Section 2: Organization characteristics**

1) Area of work

Academia

Private sector

NGO

Government

Other

2) Field of work

Social sciences

Exact sciences

Natural sciences

Health sciences

Applied sciences

Other

3) In terms of governance, the coordination of your organization/initiative/program is:

Local

National

Regional

International

Other

4) The scope of your organization/initiative/program is:

Local

National

Regional

International

Other

5) What are the objectives of the organization you represent?

Promotion of open science in the region

Development of tools of open access (hardware, software, others)

Education in open science principles

Advise on the implementation of open science strategies

Development of science outreach and/or citizen science activities

Developing open science policies

All of the above

Other

6) Does the organization have legal representation? Y/N

**Section 3: Open science conceptualization**

1) How would you define open science? (250 words maximum)

2) Do you consider this definition of open science global and applicable everywhere and by/for all people? Justify answer (250 words maximum)

3) Which of the following aspects do you consider to be part of open science?

Open access to scientific journals

Open access to data

Open software

Open hardware/infrastructure

Open access to educational resources

Open evaluation

Scientific dissemination and communication

Citizen science

All of the above

Other

4) In your experience, what benefits can Open Science bring to your work/field of research? Choose the 5 options that have the highest priority

Increases visibility and access to scientific journals

Allows researchers to access new data

Facilitates the establishment of new collaborations

Promotes reuse of data in a reliable manner

Promotes more citations of scientific articles

Increases the credibility of scientific work

Increases reproducibility of scientific work

Increases the efficiency of scientific work

Redistribution of resources

Reduction of costs associated with data production

Equality of opportunity in access to information

Provides resources for education/training activities

Changes in traditional academic hierarchies

Provides avenues of communication with the general public

Other

5) Are you concerned about any potential negative impacts of Open Science practices in your field of work/research? Y/N

6) If you answered "Yes", please select the negative impacts related to the Open Science movement that you are concerned about. Choose the 5 options that have the highest priority.

Impact factor in scientific journals that practice open science.

Data or article quality

Data sovereignty and its relation to scientific appropriation

Additional administrative steps to publish in open science journals

Reduction of scientific innovation

Extra time needed to learn open science tools and best practices

Credit/acknowledgment/copyrights

Patents

Privacy of sensitive data, e.g. health data

Lack of restrictions on sensitive projects, e.g. bioweapons, artificial intelligence for security, etc.

Errors are recorded forever

Possibility of plagiarism

**Section 4: Science diplomacy initiatives and interaction with other stakeholders**

1) In your organization, have you organized joint events with organizations from other countries? Y/N

2) If you selected "Yes", which ones? Please write in list format or separated by comma (maximum 250 words)

3) Have you applied to calls for proposals or projects at the national level and/or in other countries? Y/N

4) If "Yes", which ones? Please write in list format or separated by comma (maximum 250 words)

5) Has the organization received support from the government of your country? Y/N

6) If "Yes", how have they been supported? Please write in list format or comma-separated (maximum 250 words)

7) Has the organization been actively involved in science policies related to open science in the country, region or globally?

8) If you selected "Yes", how? Please write in list format or separated by comma (maximum 250 words)

9) Do your organization's programs/activities/actions implicitly or explicitly aim to ensure access to resources to improve the country's and/or region's innovation capabilities and competitiveness?

10) If you selected "Yes", please select all the methods you use for this purpose:

Training/education activities

Establishment of regional and/or international partnerships

Attraction of foreign talent and investment

Access to facilities/hardware

Access to software

Development of open access repositories

Open science research and innovation

Increasing localization and contextualization of material/resources

11) Do your organization's programs/activities/actions implicitly or explicitly aim to promote Open Science best practices and initiatives developed in your country and/or region at the international level? Y/N

12) If "Yes", how? Please write in list format or separated by comma (maximum 250 words)

13) Do your organization's programs/activities/actions implicitly or explicitly aim to influence the opinion of the public, your colleagues or public policy developers on the adoption of open science principles? Y/N

14) If yes, how? Please write in list format or comma separated (maximum 250 words).

15) In your experience, what are the main barriers/challenges your organization has faced in the activities described in the previous sections? Prioritize the 5 most relevant

Financing

Material available in another language

Access to computer resources

Cultural resistance in institutions

Open science is not a metric of recognition/career progression

Confidentiality

Lack of time

Internet quality

Other

**Section 5: Other stakeholders in the region**

1) Do you know of other projects/initiatives/programs/institutions working on collaborative Open Science projects in Latin America?

2) If "Yes", which ones?

3) Do you have a website/contact/social network? Please write in list format or separated by comma
